# Transcriptome Analysis and Differential Gene Expression on the Testis of Orange Mud Crab, *Scylla olivacea*, during Sexual Maturation

**DOI:** 10.1371/journal.pone.0171095

**Published:** 2017-01-30

**Authors:** Khor Waiho, Hanafiah Fazhan, Md Sheriff Shahreza, Julia Hwei Zhong Moh, Shaibani Noorbaiduri, Li Lian Wong, Saranya Sinnasamy, Mhd Ikhwanuddin

**Affiliations:** 1 Institute of Tropical Aquaculture, Universiti Malaysia Terengganu, Kuala Terengganu, Terengganu, Malaysia; 2 School of Fisheries and Aquaculture Sciences, Universiti Malaysia Terengganu, Kuala Terengganu, Terengganu, Malaysia; 3 Institute of Marine Biotechnology, Universiti Malaysia Terengganu, Kuala Terengganu, Terengganu, Malaysia; Xiamen University, CHINA

## Abstract

Adequate genetic information is essential for sustainable crustacean fisheries and aquaculture management. The commercially important orange mud crab, *Scylla olivacea*, is prevalent in Southeast Asia region and is highly sought after. Although it is a suitable aquaculture candidate, full domestication of this species is hampered by the lack of knowledge about the sexual maturation process and the molecular mechanisms behind it, especially in males. To date, data on its whole genome is yet to be reported for *S*. *olivacea*. The available transcriptome data published previously on this species focus primarily on females and the role of central nervous system in reproductive development. *De novo* transcriptome sequencing for the testes of *S*. *olivacea* from immature, maturing and mature stages were performed. A total of approximately 144 million high-quality reads were generated and *de novo* assembled into 160,569 transcripts with a total length of 142.2 Mb. Approximately 15–23% of the total assembled transcripts were annotated when compared to public protein sequence databases (i.e. UniProt database, Interpro database, Pfam database and *Drosophila melanogaster* protein database), and GO-categorised with GO Ontology terms. A total of 156,181 high-quality Single-Nucleotide Polymorphisms (SNPs) were mined from the transcriptome data of present study. Transcriptome comparison among the testes of different maturation stages revealed one gene (beta crystallin like gene) with the most significant differential expression—up-regulated in immature stage and down-regulated in maturing and mature stages. This was further validated by qRT-PCR. In conclusion, a comprehensive transcriptome of the testis of orange mud crabs from different maturation stages were obtained. This report provides an invaluable resource for enhancing our understanding of this species’ genome structure and biology, as expressed and controlled by their gonads.

## Introduction

Orange mud crab, *Scylla olivacea* is widely distributed along the equator and predominantly found in the Southeast Asia region [1–5]. It is considered as one of the most economically important marine crustacean species in Southeast Asean countries including Malaysia, Thailand, Philippines and Indonesia [2,6,7]. Until now, landing of *S*. *olivacea* around Southeast Asia region depends solely on wild fisheries and although small scale aquaculture productions were reported [2], they often only involve fattening of wild-caught marketable-sized crabs with low flesh content or production of soft-shelled crabs from captured juvenile crabs. The over-exploitation of wild *S*. *olivacea* resources, coupled with habitat loss and pollution, negatively affect its population health and indirectly impact the livelihood of coastal communities as well. One of the ways to help safeguard the natural resources of *S*. *olivacea* is to meet the market’s demand with farmed animals. In 2014, the estimated world aquaculture production of *Scylla* species was approximately 183,000 tonnes (*Scylla serrata* Fact Sheet, Cultured Aquatic Species Information Programme, Fisheries and Aquaculture Department, Food and Agriculture Organization of the United Nations; http://www.fao.org/fishery/species/2637/en [accessed February 20, 2016]). Unfortunately, most of these productions still rely on wild broodstocks and juveniles [2]. Full involvement of *S*. *olivacea* in aquaculture is currently still not possible due to the lack of in-depth knowledge in many fields, especially regarding its basic reproductive biology and physiology.

Directly related to sexual maturation and reproduction, testis is responsible for the production of male gametes via spermatogenesis and androgenic hormones. The morphology and ultrastructure of testis and germ cells of *Scylla* spp., and their histological changes during sexual maturation has been described in detail by Anbarasu et al. [8] and Waiho et al. [9], yet the regulatory mechanism and gene expression in testis during sexual maturation are still poorly understood. Extremely limited molecular studies were conducted on *S*. *olivacea* [10,11]. Most studies focus primarily on the maturation of females and tissue-specific gene expression profiles in male *S*. *olivacea* are currently unavailable [11]. The limited genome and transcriptome information available for this economically important portunid species hampers the large-scale aquaculture of *S*. *olivacea*, especially in the field of broodstock selection and artificial seed production.

Transcriptome analysis is able to reveal genes that are being actively expressed in specific tissue and species of interest, and also facilitate the discovery of potential molecular markers. This is in particular useful in non-model organisms where the full genome data is still not available for comparison [12–14]. The use of transcriptome analysis has been reported in several economically important aquaculture species [15–18]. The reproduction-related genes of commercially important crustacean species, such as swimming crab *Portunus trituberculatus*, Chinese mitten crab *Eriocheir sinensis*, green mud crab *Scylla paramamosain* and Oriental river prawn *Macrobrachium nipponense* were successfully identified via transcriptome sequencing [12, 19–21]. To date, the sequencing of whole genome and research involving next-generation sequencing of *S*. *olivacea* has yet to be reported. The availability of sufficient genome or transcriptome data are potentially useful for studies on differential gene expressions, gene regulatory mechanisms, and molecular marker application. Present study presents a comprehensive analysis of the transcriptome data derived from testis tissue of *S*. *olivacea* in different maturation stages using Illumina HiSeq. An annotated *S*. *olivacea* testis transcriptome library was constructed via *de novo* assembly of sequenced reads. The findings in this study provide an in-depth insight to the changes occurring in the testis of *S*. *olivacea* at molecular and genomic level, and could further facilitate future studies on specific functional genes, identification of molecular markers and the construction of detailed genetic map in this species.

## Materials and Methods

### Sample collection

Male *S*. *olivacea* (carapace width range = 60.0 to 123.0 mm) were obtained from Setiu Wetlands, Terengganu, Malaysia (5°38'19''N; 102°46'20''E) during July 2014. Setiu Wetlands is a common fishing ground and no licensing was required for the acquisition of mud crabs. We adhered to the ASAB (2012) “*Guidelines for the treatment of animals in behavioural research and teaching*” published in Animal Behaviour 83: 301–309. None of the work involved endangered or protected species. All crab handling and experimental procedures were approved by the Ethics Committee of Institute of Tropical Aquaculture, Universiti Malaysia Terengganu in accordance with the “Malaysian code of practice for the care and use of animals for scientific purposes” outlined by Laboratory Animal Science Association of Malaysia. All crabs were transported live back to marine hatchery of Institute of Tropical Aquaculture, Universiti Malaysia Terengganu, Terengganu, Malaysia, disinfected and maintained briefly in filtered sea water before being sacrificed.

### RNA extraction and cDNA library preparation

Crabs were categorised into three maturation stages, i.e. immature, maturing and mature, based on their gonadosomatic index (GSI) and gonad external morphologies: immature—GSI = <0.15, vas deferens are translucent and barely visible; maturing—GSI = <0.36, vas deferens are visible, milky white but not enlarged; mature—GSI = >0.40, vas deferens are milky white and swollen [9]. Testes of crabs from all maturation stages were removed and snap frozen in liquid nitrogen, with six samples per stage. Testes were homogenized using mortar and pestle and temperature was maintained low using liquid nitrogen. RNA extraction using Direct-zol^™^ RNA MiniPrep (Zymo Research, U.S.A) was conducted independently on one sample from each tissue to ensure that RNA extraction method used was able to extract sufficient quantity of high quality RNA. Subsequently, equal amount (25 mg) of the remaining homogenized samples were pooled according to maturation stage (five samples per stage) and total RNA was extracted for each pooled samples. The RNA quality and quantity were assessed using NanoDrop 2000 (Thermo Fisher Scientific Inc., USA) and Qubit 2.0 RNA Broad Range Assay (Invitrogen, USA) respectively. The RNA integrity number (RIN) of each samples were measured using Agilent Bioanalyzer (Agilent, USA). All samples were selected for sequencing (RIN in the range of 7.4–8.3). RNA were then pooled according to maturation stages.

mRNA isolation and cDNA synthesis were performed using NEBNext^®^ Ultra^™^ RNA Library Prep Kit for Illumina^®^ according to manufacturer’s protocol. The synthesized cDNA was quantified using Qubit 2.0 DNA Broad Range Assay (Invitrogen, USA). A minimum of 10ng cDNA was fragmented using Covaris S220 (Covaris Inc, USA) to a targeted size of 200–300 bp. The fragmented cDNAs were then end-repaired, ligated to NEBNext adapters, and PCR-enriched using NEBNext^®^ Ultra^™^ RNA Library Prep Kit. The final sequencing libraries were quantified using KAPA kit (KAPA Biosystem, USA) on Agilent Stratagene Mx-3005p quantitative PCR (Agilent, USA) and sizes were confirmed using Agilent Bioanalyzer High Sensitivity DNA Chip (Agilent, USA). The resulting sequencing libraries were sequenced using an Illumina flow cell, and 209 cycles on the Illumina HiSeq^™^ 2000 platform (Illumina, USA). The sequencing run generated a total of 17 GB of raw data.

### Pre-processing and *de novo* assembly

Adapter clipping, trimming reads based on quality, and removing sequences with ambiguous bases (N) was conducted using Trimmomatic version 0.32 [22] and Prinseq-lite version 0.20.4 [23]. FastQC assessment reports of sequence reads were used to evaluate read quality before and after pre-processing. All subsequent analyses were conducted using clean reads.

After pre-processing, the clean reads from the data sets were assembled by *de novo* assembly using Trinity RNA-Seq version 2.0.4 [24]. Reference transcripts were generated by combining all clean reads of the Illumina sequencing data sets. Only one gene (the longest one) was selected to represent the assembled component from each cluster to prevent redundancy [24]. Transcriptome assembly completeness was analysed using BUSCO [25] against a set of 2,675 arthopoda genes to evaluate the quality of the final assembly. All clean reads of *de novo* assembly sequence data from *S*. *olivacea* were deposited in GenBank, National Centre for Biotechnology Information (NCBI, USA, http://ww.ncbi.nlm.nih.gov/) under the Accession No. GDRN00000000 (BioProject Accession No. PRJNA289610).

### Functional annotation

Homology searches and assembled transcripts mapping were conducted using Blastx (version: ncbi-blast-2.2.30+) against the UniProt database, Interpro database, Pfam (Protein family) database and *Drosophila melanogaster* protein database with a cut-off e-value of 1e-5. The top (best) hit from each assembled transcript comparisons were used as the annotation reference for the respective transcripts. The Gene Ontology (GO) terms of *S*. *olivacea* were further analysed using Blast2GO software v.2.6.0 [26,27] based on default parameters (e-value < 1e-6, annotation cut-off > 55 and a GO weight > 5).

### Single Nucleotide Polymorphism (SNP) calling

For SNPs calling, only reliable, Bowtie mapped reads were considered. Insertion or deletion variations (InDels) were excluded because alternative splicing impedes reliable InDel discovery. SNPs were called using SAMtools mpileup [28]. Genotype likelihoods were computed using SAMtools utilities. Variable positions in the aligned reads were compared to the reference transcripts using the BCFtools utilities. Read depth ≥ 10, SNP reads/total reads ratio ≥ 25, SNP quality ≥ 50 and mapping quality ≥ 20 were used to filter false positive SNPs by using in-house Perl scripts.

### Identification and validation of differentially expressed gene

To identify differentially expressed genes, paired-end reads were first aligned back to the assembled transcripts (length ≥ 300 bp) using RSEM [29]. Transcripts’ abundance was then estimated and alternatively-spliced transcripts were constructed. In some rare cases, these transcripts may be from paralogs that shared high sequence similarity. Differential expression analysis between samples was conducted using edgeR [30]. Expected counts of mapped read pairs were normalized, and the fold changes and p-values for each gene or transcript were calculated. Results were then filtered based on a set of threshold values (log2FoldChange and adjusted P- (P_adj_) value < 0.05). For the identification of significantly differentially expressed genes, only genes with padj value of < 1e-10 was considered.

Total RNA from immature, maturing and mature specimens were extracted using Direct-zol^™^ RNA MiniPrep (Zymo Research, U.S.A) and converted to cDNA using iScript^™^ Reverse Transcription Supermix (Bio-Rad, USA) as per manufacturer’s protocol. Approximately 5 μl of RNA served as template for cDNA conversion and the incubation protocol was: priming at 25°C for 5 min, reverse transcription at 42°C for 30 min and inactivation at 85°C for 5 min. Quantitative real-time polymerase chain reaction (qPCR) was run in Miniopticon Real-time PCR system (Bio-Rad, USA) with SYBR Green PCR Master Mix (Bio-Rad, USA) to validate differentially expressed genes obtained from transcriptome data. Primers were designed using PrimerQuest Tool (Integrated DNA Technologies Inc., Singapore) with housekeeping gene 18S rRNA [31] as internal control (normalization gene) (Table 1). Three biological replicates and two technical replicates for each maturation stage were run along with internal control in qPCR. Standard manufacturer protocol was applied, with each qPCR reaction (total volume = 25 μl) contained 10 ng cDNA as template. The temperature profile used was initial denaturation at 95°C for 3 min, followed by 40 cycles of denaturation at 95°C for 15 s and annealing at 60°C for 30 s. cDNA template was replaced with diethylpyrocarbonate water in negative control. Comparative Cycle Threshold (C_T_) method [32] was used to determine the fold difference of studied gene in different maturation stages. One-Way ANOVA was used to determine statistical difference between maturation stages (significant value at *p* < 0.05), followed by Tukey’s test. All statistical analyses were conducted using Microsoft excel 2013.

**Table 1:** Primers used in quantitative real-time polymerase chain reaction.

Gene name	Primers	Sequence	Target size	Reference
Beta crystallin like gene	BCG-F	5’-GCATGTACCCAGAACGGAGT-3’	103 bp	-
	BCG-R	5’-TTTACCACAAGCTGCTGCAC-3’		
18S rRNA	qRT-F	5’-ATGATAGGGATTGGGGTTTGC-3’	-	Wang et al. [31]
	qRT-R	5’-AGAGTGCCAGTCCGAAGG-3’		

## Results

### Transcriptome sequencing and read assembly

**Fig 1:**
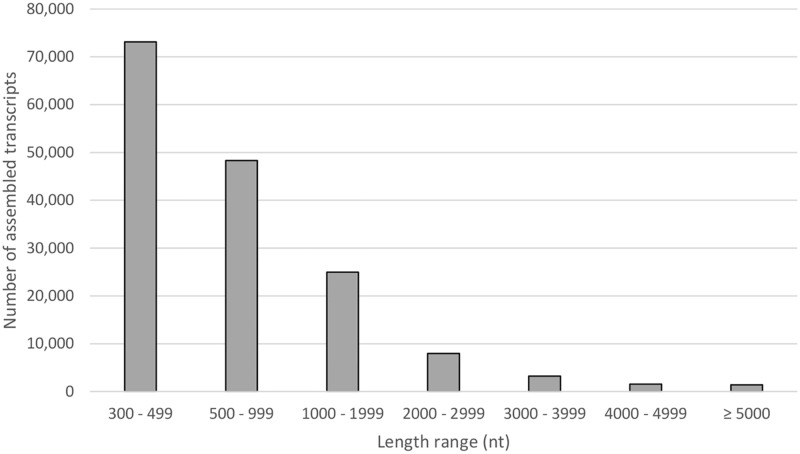
Graphical length distribution summary of transcripts identified in *S*. *olivacea* transcriptome data sets.

Three cDNA libraries representing different maturation stages (i.e. immature, maturing and mature) of *S*. *olivacea* were sequenced using Illumina HiSeq 2000 platform. A total number of 76,337,338, 64,928,802 and 30,841,304 raw reads were obtained from immature, maturing and mature male crabs respectively. Approximately 86.27%, 86.05% and 74.02% of clean reads were retrieved after pre-processing (adaptor removal, quality trimming and N removals) to discard low quality and empty reads (Table 2). A large number of reads (86.50%) aligned back to the transcripts as expected (Table 2). Reads that did not map back to the assembled transcripts corresponded to either low quality reads or lowly-expressed transcripts that could not be assembled due to the minimum length requirement (≥ 300 nt). The assembled transcripts (n = 160,569) had a total size of 142,192,028 bp, an average size of 886 bp, assembled transcript range of 300 bp to 16,041 bp and a N50 assembled transcripts length of 1,225 (Table 2). Nearly half of (45.55%) of the assembled transcripts were at the length range of 300–499 nt (Fig 1). Approximately 41% (n = 64,793) of assembled transcripts contained protein-coding potential. Busco analysis revealed that 2,045 out of 2,675 genes could be fully annotated (76% completeness) and 2,355 out of 2,645 genes met the criterion for partial annotation (88.04% completeness).

**Table 2:** Summary of assembly statistics.

	Immature crabs	Maturing crabs	Mature crabs
**Raw sequencing reads**			
Total reads	76,337,338	64,928,802	30,841,304
Total bases (bp)	7,710,071,138	6,557,809,002	3,114,971,704
**Clean sequencing reads**			
Total reads	65,859,364	55,873,266	22,828,074
Total bases (bp)	6,578,177,998	5,579,213,298	2,261,133,698
Percentage of clean reads (%)	86.27	86.05	74.02
Percentage of clean bases (%)	85.32	85.08	72.59
**Alignment statistics**			
Total Reads	144,560,704		
Reads Aligned	125,050,327		
% Reads Aligned	86.50		
Assembled Transcripts Length (in bases)	142,192,028		
Total Assembled Transcripts Covered (in bases)	140,431,613		
% Total Assembled Transcripts Covered	98.76		
Average Read Depth	73.39		
**Assembled statistics**			
Number of assembled transcripts	160,569		
Total size of assembled transcripts (bp)	142,192,028		
Longest assembled transcripts (bp)	16,041		
Shortest assembled transcripts (bp)	300		
Number of assembled transcripts > 1K nt	39,060		
Number of transcripts > 10K nt	49		
Mean assembled transcripts size	886		
N50 assembled transcripts length	1,225		
Assembled transcripts %A	26.87		
Assembled transcripts %C	22.96		
Assembled transcripts %G	23.86		
Assembled transcripts %T	26.31		
Assembled transcripts %N	0		
Assembled transcripts %non-ACGTN	0		

### Functional annotation

**Fig 2:**
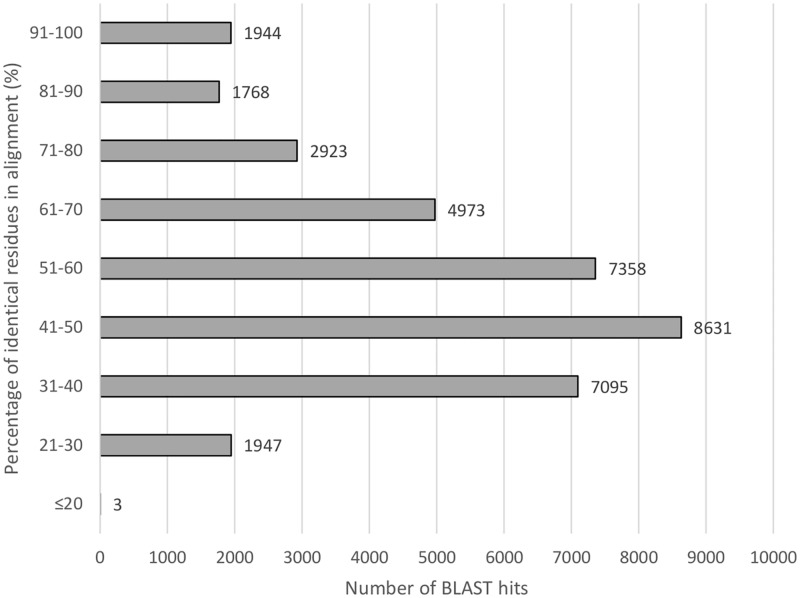
Similarity distribution of BLAST hits.

**Fig 3:**
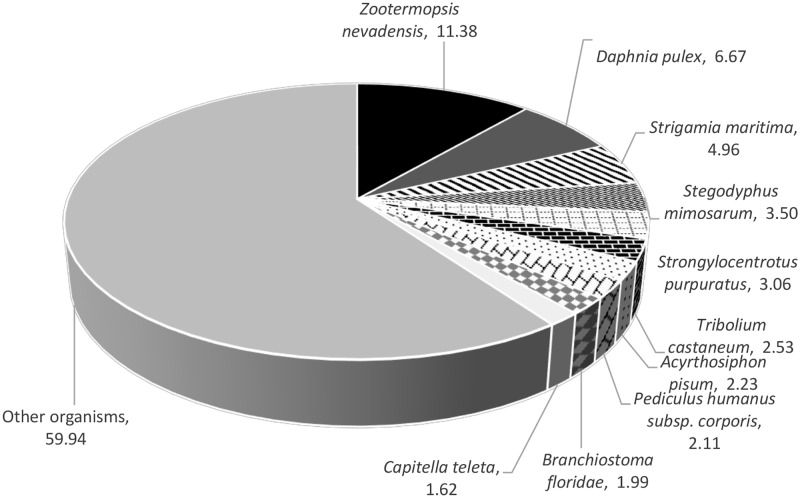
Species distribution of top ten BLAST hits against UniProt database and *D*. *melanogaster* protein database, with a 1e-5 cut-off threshold.

BLASTx search against the UniProt database, Interpro database, Pfam database and *D*. *melanogaster* protein database was conducted to annotate the consensus sequences. Out of 160,569 total number of assembled transcripts, 36,642 (22.82%) transcripts mapped back to UniProt database, 25,511 (15.89%) transcripts mapped back to Interpro database, 23,620 (14.71%) transcripts mapped back to Pfam database and 25,375 (15.80%) transcripts mapped back to *D*. *melanogaster* protein database (1e-5 cut-off threshold). A total of 240 transcripts (0.95%) to the *D*. *melanogaster* protein database were full length. Approximately 75.32% of the top-hit alignments had a similarity of higher than 40% (Fig 2). Seven out of the top ten organism hits in *S*. *olivacea* transcriptome against UniProt database were Arthropods (Table 3). Nevada termite, *Zootermopsis nevadensis* had the highest matched assembled transcripts percentage (11.38%) followed by water flea, *Daphnia pulex* (6.67%) and European centipede, *Strigamia maritima* (4.96%) (Fig 3). Among the annotated transcripts, 480, 56, 8 and 1 transcripts were similar to that of other *Scylla* species in UniProt database, i.e. *S*. *paramamosain*, *S*. *serrata*, *S*. *olivacea* and *S*. *tranquebarica* respectively. The top 20 high quality annotations of *S*. *olivacea* transcriptome based on E value and bit score are listed in Table 4.

**Table 3:** Top 10 organism hits of assembled transcripts in *S*. *olivacea* transcriptome against UniProt database.

Organism scientific name (common name)	Taxonomy			Matched assembled transcripts (n)
	Phylum	Subphylum	Class	
*Zootermopsis nevadensis* (Nevada termite)	Arthropoda	Hexapoda	Insecta	4,170
*Daphnia pulex* (Water flea)	Arthropoda	Crustacea	Branchiopoda	2,444
*Strigamia maritima* (European centipede)	Arthropoda	Myriapoda	Chilopoda	1,817
*Stegodyphus mimosarum* (Spider)	Arthropoda	Chelicerata	Arachnida	1,284
*Strongylocentrotus purpuratus* (Purple sea urchin)	Echinodermata	Echinozoa	Echinoidea	1,123
*Tribolium castaneum* (Red flour beetle)	Arthropoda	Hexapoda	Insecta	927
*Acyrthosiphon pisum* (Pea aphid)	Arthropoda	Hexapoda	Insecta	816
*Pediculus humanus subsp*. *Corporis* (Body louse)	Arthropoda	Hexapoda	Insecta	774
*Branchiostoma floridae* (Florida lancelet)	Chordata	Cephalochordata	Leptocardii	731
*Capitella teleta* (Polychaete worm)	Annelida	-	Polychaeta	593

**Table 4:** Top 20 annotations of *S*. *olivacea* transcriptome with the highest bit score.

Description	Accession ID	Organism Scientific name (common name)	Alignment length (amino acids)	E value	Bit Score	Type
Cj-cadherin	Q5CCS4	*Caridina multidentata* (Amano shrimp)	3007	0	5472	Full length
Dynein heavy chain, cytoplasmic	A0A067RE92	*Zootermopsis nevadensis* (Nevada termite)	3298	0	5434	Full length
Uncharacterized protein	K7J7S2	*Nasonia vitripennis* (Jewel wasp)	4156	0	5202	Partial
Projectin	Q86GD6	*Procambarus clarkii* (Red swamp crayfish)	2694	0	4756	Partial
Pre-mRNA-processing-splicing factor, putative	E0VM49	*Pediculus humanus* subsp. *corporis* (Body louse)	2373	0	4354	Full length
Uncharacterized protein	T1JAK1	*Strigamia maritima* (European centipede)	3404	0	4342	Partial
Spectrin alpha chain	A0A067RUI8	*Zootermopsis nevadensis* (Nevada termite)	2422	0	4045	Full length
Laminin subunit alpha	A0A067R415	*Zootermopsis nevadensis* (Nevada termite)	3616	0	3516	Full length
Talin-1	A0A067R9F3	*Zootermopsis nevadensis* (Nevada termite)	2522	0	3336	Partial
Spectrin beta chain	A0A067R2J7	*Zootermopsis nevadensis* (Nevada termite)	2151	0	3322	Full length
Ciliary dynein heavy chain, putative	E0VLA6	*Pediculus humanus subsp*. *corporis* (Body louse)	2193	0	3212	Partial
Myosin Va	F6K356	*Eriocheir sinensis* (Chinese mitten crab)	1778	0	3203	Full length
Putative uncharacterized protein	E9G1C9	*Daphnia pulex* (Water flea)	2247	0	3094	Full length
Putative uncharacterized protein	D6X207	*Tribolium castaneum* (Red flour beetle)	2254	0	3049	Full length
Dynein beta chain, ciliary	P39057	*Heliocidaris crassispina* (Sea urchin)	2780	0	3025	Partial
Clathrin heavy chain	A0A067RP81	*Zootermopsis nevadensis* (Nevada termite)	1683	0	2978	Full length
Target of rapamycin	B5M076	*Blattella germanica* (German cockroach)	2495	0	2963	Full length
Fatty acid synthase	F8RHR0	*Litopenaeus vannamei* (Whiteleg shrimp)	2445	0	2928	Partial
DNA-directed_RNA_polymerase	V5YTD8	*Oratosquilla oratoria* (Japanese mantis shrimp)	1552	0	2916	Full length
Putative U5 small nuclear ribonucleoprotein 200 kDa helicase	A0A067RJY2	*Zootermopsis nevadensis* (Nevada termite)	1891	0	2883	Partial

**Fig 4:**
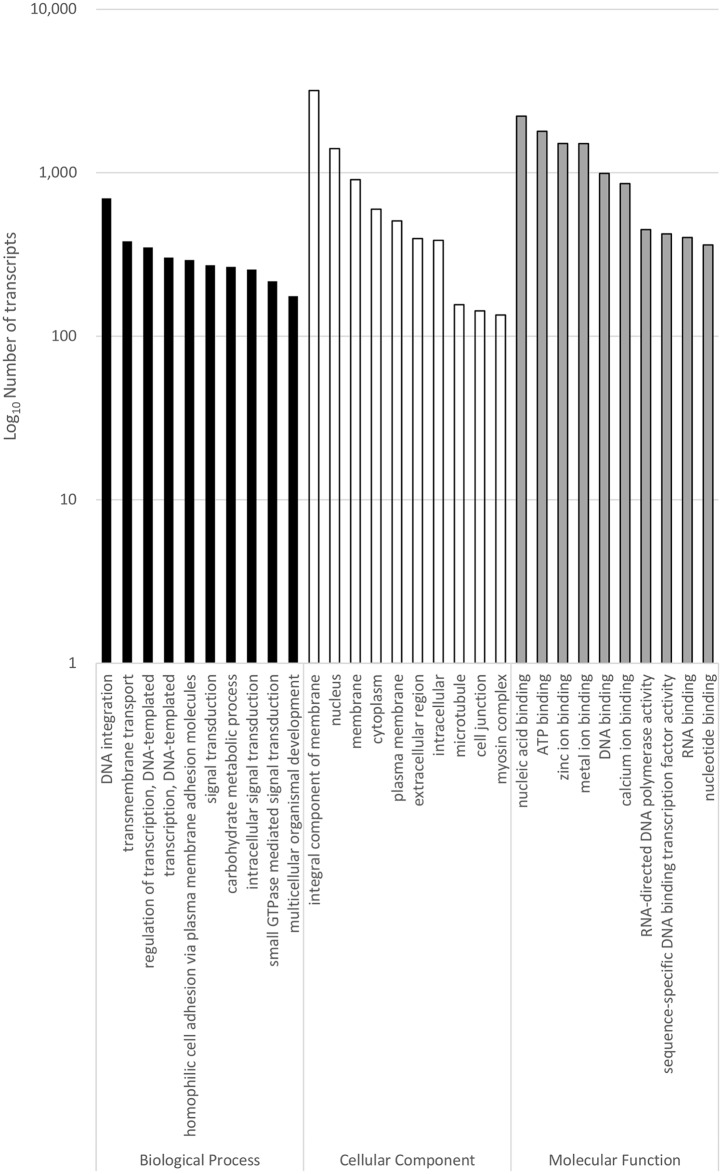
Gene ontologies. Transcript counts (log_10_) for Gene Ontology (GO) classification of the *S*. *olivacea* transcriptome for biological process (black), cellular component (white) and molecular function (grey) categories.

GO terms of *S*. *olivacea* transcriptome were analysed using the GO classification system. A total of 19,155 (52%) transcripts were GO-categorized into one of the three GO domains, i.e. biological process (12,250 transcripts), cellular component (11,129 transcripts) and molecular function (26,805 transcripts) while the remaining 17,487 transcripts were unassigned. Fig 4 shows the distribution of transcripts across the top 10 GO terms for each of the three GO domains. The top three categories in the biological process GO domain were “DNA integration” (698 transcripts), “transmembrane transport” (381 transcripts) and “regulation of transcription, DNA-templated” (350 transcripts). In the cellular component GO domain, most of the transcripts were involved in “integral component of membrane” (3185 transcripts), “nucleus” (1406 transcripts) and “membrane” (908 transcripts). “nucleic acid binding”, “ATP binding” and “zinc ion binding” were the top three categories in the molecular function GO domain, with a total number of assigned transcripts of 2222, 1794 and 1511 respectively.

### Genes associated with growth, development and reproduction

During the annotation process, a number of GO terms associated with growth, development and reproduction processes, especially with the term from the ontology of “multicellular organismal development” (GO:0007275). The child terms and co-occurring terms associated with this parent category are listed in Table 5. The regulators (i.e. proteins) of growth, development and reproduction were identified from *S*. *olivacea* transcriptome annotation results (Table 6).

**Table 5:** Number of hits of selected GO child terms and co-occurring terms based on multicellular organismal development (GO:0007275).

GO ID	GO term	Hits	Example Sequence	E value	Accession ID
*Child Terms*					
GO:0007349	Cellularization	2	Vielfältig, isoform D	9e-10	M9NES1
GO:0007566	Embryo implantation	2	Uncharacterized protein	1e-73	F6SMD1
GO:0009790	Embryo development	2	Trip12 protein	1e-139	Q3KR60
GO:0009791	post-embryonic development	1	Uncharacterized protein	1e-21	ALDH5A1
GO:0030237	Female sex determination	1	Protein Wnt	1e-27	WNT4
GO:0030238	Male sex determination	1	Tyrosine-protein kinase receptor	4e-139	IGF1R
*Co-occurring terms*					
GO:0007283	Spermatogenesis	11	Gilgamesh isoform E	0	Q59DW8
GO:0010468	Regulation of gene expression	2	Putative uncharacterized protein	1e-43	Q8CDC6
GO:1900194	Negative regulation of oocyte maturation	2	Uncharacterized protein	1e-10	F6Q3S2
GO:1902436	Negative regulation of male mating behaviour	1	Putative uncharacterized protein	1e-43	Q8CDC6
GO:0061369	Negative regulation of testicular blood vessel morphogenesis	1	Protein Wnt	1e-27	F6UNR8
GO:0051781	Positive regulation of cell division	10	PDGF-and VEGF-related factor	6e-10	V9IG02
GO:0050793	Regulation of developmental process	5	Notch protein	0	A0MK40
GO:0040014	Regulation of multicellular organism growth	3	Amyloid beta protein	8e-63	M1EDY4
GO:0040034	Regulation of development, heterochronic	2	Hunchback transcription factor	2e-114	C4PGG7
GO:0048047	Mating behaviour, sex discrimination	1	Putative uncharacterized protein	1e-43	Q8CDC6
GO:0007617	Mating behaviour	1	Amyloid beta protein	8e-63	M1EDY4

**Table 6:** Selected regulators (i.e. proteins) of growth, sexual differentiation and reproduction expressed in the testis of *S*. *olivacea*.

Identity	Accession ID	Hit organism	Similarity (%)	E value	Example Query ID
*Neurohormones*					
Vitellogenesis-inhibiting hormone (VIH)	V9ZBG5	*Scylla paramamosain* (Green mud crab)	99.2	5e-85	Ref_Crab_Transcript_38360_732
Crustacean hyperglycemic hormone (CHH)	A5A599	*Scylla olivacea* (Orange mud crab)	99.28	2e-91	Ref_Crab_Transcript_59696_1668
Neuropeptide	B4IC30	*Drosophila sechellia* (Fruit fly)	51.43	2e-11	Ref_Crab_Transcript_54220_1201
Neurotrophin	G5CJW4	*Litopenaeus vannamei* (Whiteleg shrimp)	68.57	2e-53	Ref_Crab_Transcript_58755_445
Neuroparsin	A0A023PY98	*Metapenaeus ensis* (Greasyback shrimp)	47.13	8e-12	Ref_Crab_Transcript_70043_2913
*Sexual differentiation related*					
SOX14 protein	B9VWK7	*Scylla paramamosain* (Green mud crab)	99.48	0	Ref_Crab_Transcript_45440_2864
VASA-like protein variant	E5FQX4	*Scylla paramamosain* (Green mud crab)	99.05	0	Ref_Crab_Transcript_27201_2369
Sex-lethal	V9PP85	*Eriocheir sinensis* (Chinese mitten crab)	98.71	1e-97	Ref_Crab_Transcript_49023_2008
Piwi-like protein	X2CS90	*Portunus trituberculatus* (Swimming crab)	96.65	0	Ref_Crab_Transcript_53740_1185
Doublesex and mab-3 related transcription-like protein	D7REN5	*Eriocheir sinensis* (Chinese mitten crab)	95.7	3e-56	Ref_Crab_Transcript_69520_1864
Doublesex and mab-3 related transcription factor 11E	X2D7J9	*Macrobrachium rosenbergii* (Giant freshwater prawn)	86.67	2e-12	Ref_Crab_Transcript_31999_445
Male-specific lethal 3-like protein	A0A067R1H2	*Zootermopsis nevadensis* (Nevada termite)	82.98	3e-41	Ref_Crab_Transcript_88052_3530
VASA	A0A023JMC5	*Charybdis japonica* (Asian paddle crab)	80	3e-18	Ref_Crab_Transcript_23777_903
*Growth and development related*					
Krueppel-like factor 10	Q13118	*Homo sapiens* (Human)	100	2e-79	Ref_Crab_Transcript_160024_379
Growth factor receptor-bound protein 2	U6DGZ9	*Neovison vison* (American mink)	100	4e-83	Ref_Crab_Transcript_151979_370
Transforming growth factor-beta regulator I	H9B3Y8	*Scylla paramamosain* (Green mud crab)	99.27	1e-91	Ref_Crab_Transcript_149715_1563
Sex combs reduced	A0A059PB91	*Parhyale hawaiensis* (Amphipod)	93.58	3e-48	Ref_Crab_Transcript_44496_829
Early growth response protein 3	A0A067R8D8	*Zootermopsis nevadensis* (Nevada termite)	90.71	8e-87	Ref_Crab_Transcript_152664_761
Male reproductive-related LIM protein	B8LG57	*Macrobrachium rosenbergii* (Giant freshwater prawn)	89.13	1e-33	Ref_Crab_Transcript_29874_1367
Prostaglandin E synthase 2	M1F4P3	*Penaeus monodon* (Giant tiger prawn)	78.92	0	Ref_Crab_Transcript_59927_1959
Prostaglandin F synthase	M1F418	*Penaeus monodon* (Giant tiger prawn)	77.92	1e-170	Ref_Crab_Transcript_36654_1601
Up-regulated during skeletal muscle growth protein 5	A0A067QSY5	*Zootermopsis nevadensis* (Nevada termite)	69.23	2e-14	Ref_Crab_Transcript_21795_733
Fibroblast growth factor receptor substrate 2	A0A067RCV1	*Zootermopsis nevadensis* (Nevada termite)	66.67	3e-40	Ref_Crab_Transcript_50850_3332
Putative transforming growth factor beta receptor 1	L7MGX7	*Rhipicephalus pulchellus* (Zebra tick)	63.67	0	Ref_Crab_Transcript_59348_1873
Inhibitor of growth protein	A0A067QYK9	*Zootermopsis nevadensis* (Nevada termite)	66.91	1e-52	Ref_Crab_Transcript_62117_1271
Epidermal growth factor receptor	A0A067R240	*Zootermopsis nevadensis* (Nevada termite)	68.7	0	Ref_Crab_Transcript_84884_4296
Vascular endothelial growth factor receptor 2	A0A067QWZ0	*Zootermopsis nevadensis* (Nevada termite)	69.81	8e-16	Ref_Crab_Transcript_127316_361
*Hormone enzymes and receptors*					
Estrogen-related receptor	D2Y1A7	*Scylla paramamosain* (Green mud crab)	100	6e-22	Ref_Crab_Transcript_7743_1209
Insulin-like androgenic gland hormone	A0A075INW9	*Scylla paramamosain* (Green mud crab)	98.51	7e-39	Ref_Crab_Transcript_35719_619
Red-pigment concentrating hormone	U3PE66	*Scylla paramamosain* (Green mud crab)	97.8	1e-18	Ref_Crab_Transcript_16600_356
Bursicon hormone alpha subunit	C3S7D8	*Callinectes sapidus* (Blue crab)	96.88	6e-19	Ref_Crab_Transcript_2620_1085
E75 nuclear receptor	Q3I5Q8	*Gecarcinus lateralis* (Blackback land crab)	95.55	0	Ref_Crab_Transcript_69307_4236
Prohormone convertase	D0UJV3	*Libinia emarginata* (Longnose spider crab)	93.55	2e-9	Ref_Crab_Transcript_15118_512
Pigment dispersing hormone receptor	C6L2K2	*Penaeus japonicus* (Kuruma shrimp)	80.85	1e-16	Ref_Crab_Transcript_149701_305
Growth hormone secretagogue receptor type	A0A026WUM1	*Cerapachys biroi* (Clonal raider ant)	78.79	5e-7	Ref_Crab_Transcript_136683_553
Lutropin-choriogonadotropic hormone receptor	A0A067QJD2	*Zootermopsis nevadensis* (Nevada termite)	76.32	3e-32	Ref_Crab_Transcript_5206_458
Gonadotropin-releasing hormone receptor	A0A087TGG2	*Stegodyphus mimosarum* (Communit nest spider)	62.14	5e-55	Ref_Crab_Transcript_35602_1201
Juvenile hormone epoxide hydrolase 2	V9IEI5	*Apis cerana* (Asian honey bee)	60.42	2e-11	Ref_Crab_Transcript_96004_451
*Ecdysteroids and receptors*					
Retinoid-X receptor-2	S4TH64	*Callinectes sapidus* (Blue crab)	99.49	0	Ref_Crab_Transcript_60375_1703
Ecdysteroid receptor	O76246	*Uca pugilator* (Atlantic sand fiddler crab)	95.81	2e-47	Ref_Crab_Transcript_141583_1159
Ecdysteroid receptor 3	I6UZ31	*Scylla paramamosain* (Green mud crab)	92.94	2e-99	Ref_Crab_Transcript_46583_2256
Ecdysteroid receptor 2	I6V8K3	*Scylla paramamosain* (Green mud crab)	87.65	0	Ref_Crab_Transcript_46581_2790
Putative ecdysteroids/dopamine receptor	D6WWZ1	*Tribolium castaneum* (Red flour beetle)	59.09	4e-79	Ref_Crab_Transcript_110825_1460

### SNP discovery

**Fig 5:**
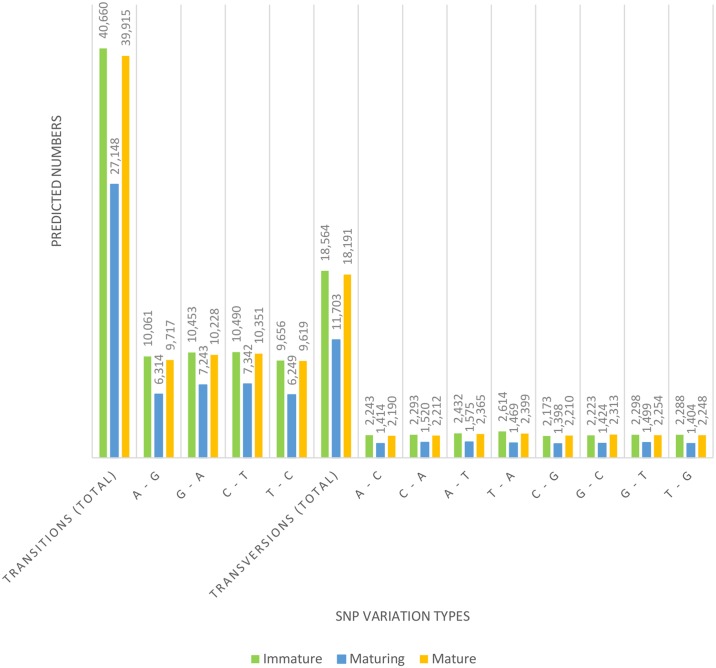
Distribution of putative single nucleotide polymorphisms (SNPs) in *S*. *olivacea* sequences.

A total of 156,181 potential SNPs (59,224 SNPs in Immature, 38,851 in Maturing and 58,106 in Mature) were identified from 481,707 transcripts (Fig 5, S1 Appendix). The transition (Ts): transversion (Tv) SNPs ratios of Immature, Maturing and Mature were 2.19: 1.00, 2.32: 1.00 and 2.19: 1.00 respectively, with a mean ratio of 2.22: 1.00. SNP types A↔G and C↔T were the most common and their numbers were similar in each maturation stage. Similar trend was observed in the numbers of transversion types A↔C, A↔T, G↔C and G↔T (Fig 5).

### Identification and validation of differentially expressed gene

**Fig 6:**
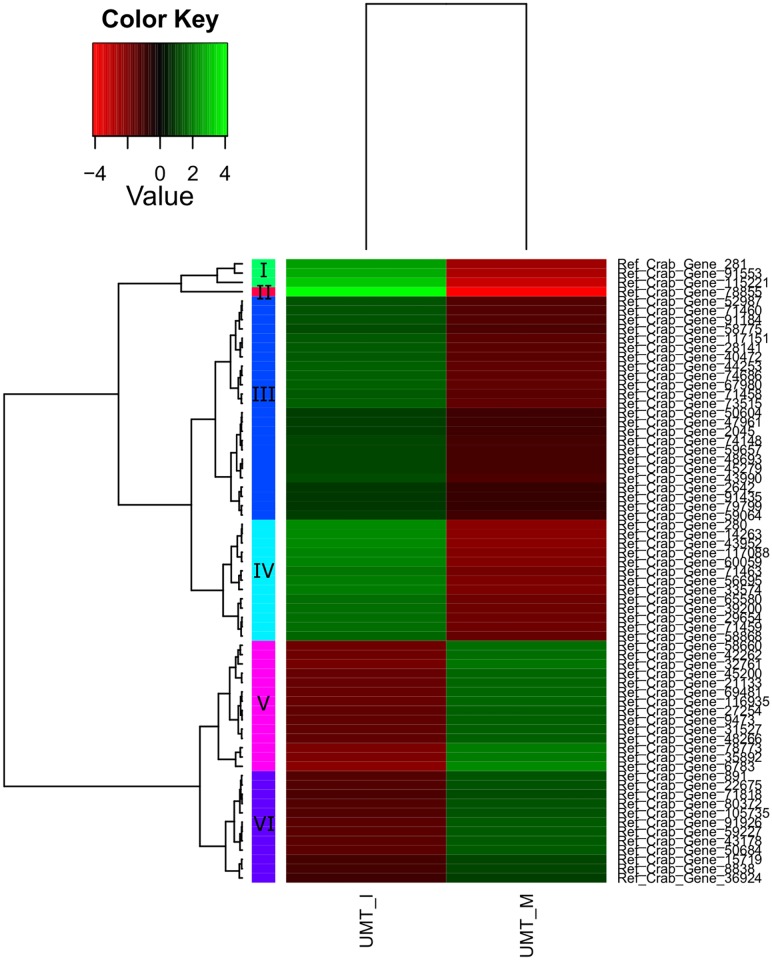
Heatmap of gene expression values depicting clustering of genes between immature (UMT_I, left panel) and mature stages (UMT_M, right panel) based on the expression of mRNAs for a set of significant genes (Padj < 0.05). Sample names are represented in columns and significant genes are represented in rows. Genes are clustered together based on expression similarity. Low to high expression is represented by a change of colour from red to green, respectively. The colour key scale bar at upper left shows Z-score values for the heatmap.

**Fig 7:**
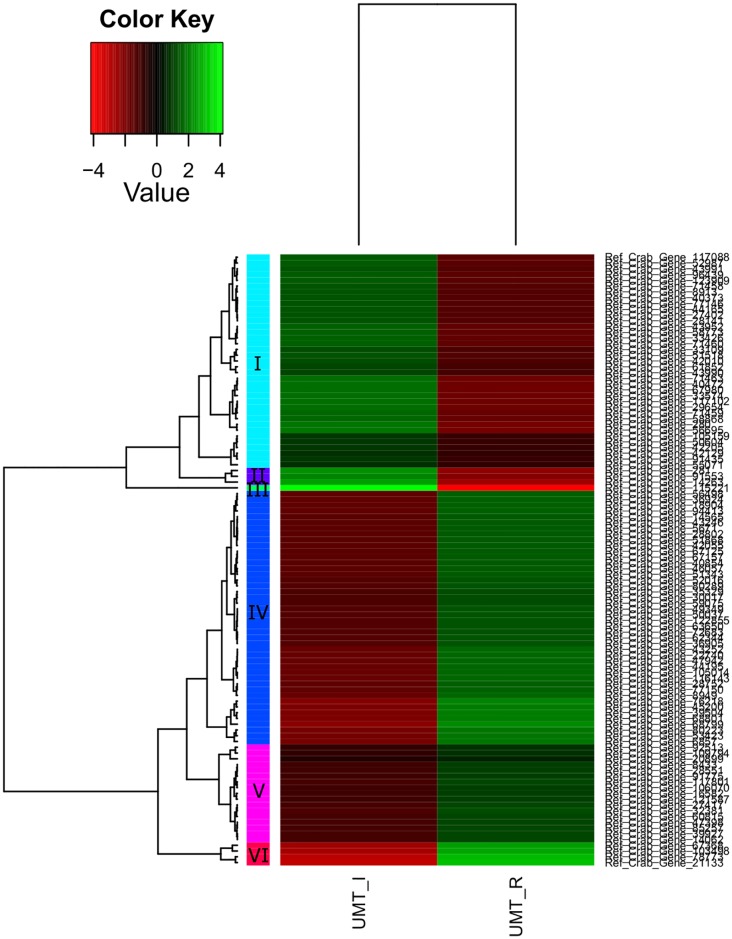
Heatmap of gene expression values depicting clustering of genes between immature (UMT_I, left panel) and mature stages (UMT_R, right panel) based on the expression of mRNAs for a set of significant genes (Padj < 0.05). Sample names are represented in columns and significant genes are represented in rows. Genes are clustered together based on expression similarity. Low to high expression is represented by a change of colour from red to green, respectively. The colour key scale bar at upper left shows Z-score values for the heatmap.

**Fig 8:**
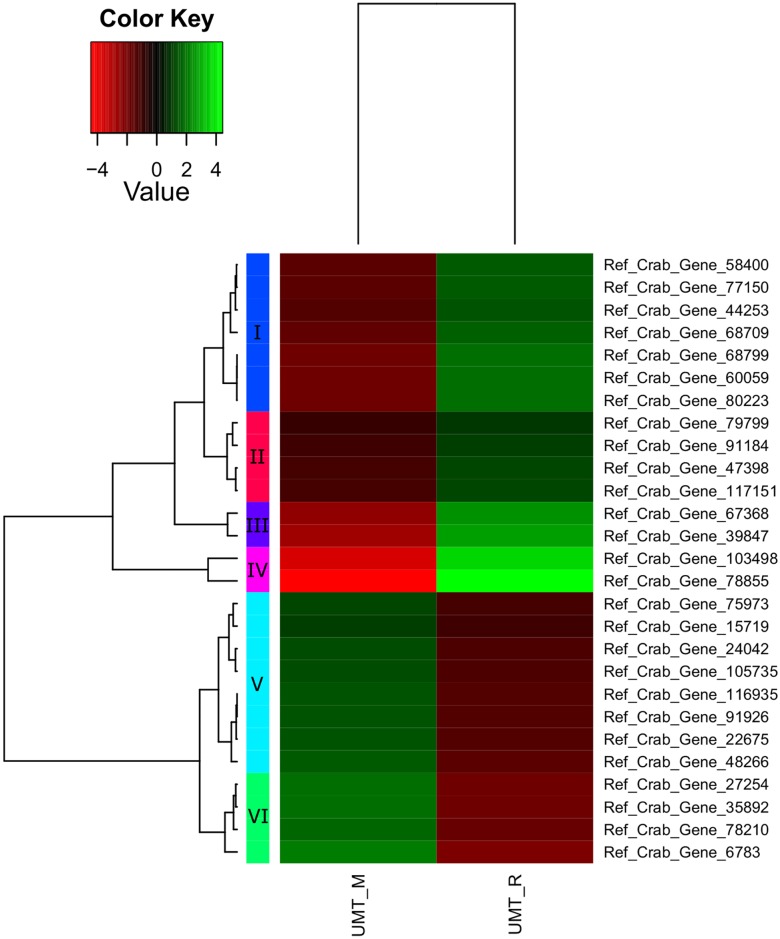
Heatmap of gene expression values depicting clustering of genes between immature (UMT_M, left panel) and mature stages (UMT_R, right panel) based on the expression of mRNAs for a set of significant genes (Padj < 0.05). Sample names are represented in columns and significant genes are represented in rows. Genes are clustered together based on expression similarity. Low to high expression is represented by a change of colour from red to green, respectively. The colour key scale bar at upper left shows Z-score values for the heatmap.

A total of 200 genes were up- or down-regulated with a P_adj_ value of < 0.05 (Table 7, S2 Appendix). Of these differentially expressed genes, only 69 genes were successfully annotated, while the remaining 65.5% are novel genes. Significant differential expression patterns between different maturation stages of *S*. *olivacea* are clearly seen in the heatmaps (Figs 6, 7 and 8). In general, more differentially expressed genes were found in the comparison involving immature crabs (67 and 106 differentially expressed genes were found for the comparison between immature and mature crabs, and between immature and maturing crabs, respectively) than the comparison between mature and maturing crabs (27 differentially expressed genes). Differentially expressed genes that were annotated (excluding genes encoding for uncharacterized proteins) are tabulated in Table 8 based on the different clustering within each heatmap. However, application of minimum threshold of Padj < 1e-10 revealed only one gene that is likely a potential candidate marker for immature crabs.

**Table 7:** Differential expression analysis of *S*. *olivacea* at gene level.

All genes					Significant differentially expressed genes (P_adj_ < 0.05)	
Cond. 1	Cond. 2	Total	Up-regulation (Cond. 2 > Cond. 1)	Down-regulation (Cond. 2 < Cond. 1)	Up-regulation (Cond. 2 > Cond. 1)	Down-regulation (Cond. 2 < Cond. 1)
I	M	121,288	53,598	67,690	26	41
I	R	125,107	62,787	62,320	65	41
M	R	115,207	62,183	53,024	15	12

Note: Cond.: condition; I: immature; M: mature; R: maturing.

**Table 8:** Selected differentially expressed genes between maturation stages in the *S*. *olivacea* testis expression profile.

Cluster on heatmap	Description	Log2FC	P_adj_	Regulation	Accession ID of Hit
**Cond.: I vs M**					
I	beta crystallin like gene	-6.78	7.74E-11	−	D3PHS5
III	Aminopeptidase	-5.00	3.46E-02	−	A0A067QSR5
III	Beta-2-microglobulin	-8.18	2.22E-03	−	P16213
I	beta crystallin like gene	-6.78	7.74E-11	−	D3PHS5
III	Aminopeptidase	-5.00	3.46E-02	−	A0A067QSR5
III	Beta-2-microglobulin	-8.18	2.22E-03	−	P16213
III	C-type lysozyme	-6.17	1.63E-03	−	B2R4C5
III	Elongation factor 1-alpha	-9.13	2.71E-05	−	W5PHA3
III	MHC class I antigen	-7.59	2.60E-02	−	R4ZGR1
III	Mobile element protein	-5.36	8.51E-03	−	L7VVN2
III	Prosaposin	-8.65	2.00E-04	−	A0A024QZQ2
III	Transposase	-5.53	4.51E-03	−	G8UKJ2
III	Transposase	-5.28	1.21E-02	−	A3JAS2
III	Transposase	-4.87	6.51E-03	−	A0A037X5S6
III	Transposase mutator type	-5.66	2.22E-03	−	R9CHU0
IV	Actin 1	-4.08	1.27E-02	−	C5HF65
IV	Gamma-crystallin A	-4.88	1.90E-05	−	D3PIA3
V	Vitellogenin	3.00	4.13E-02	+	Q9UAR3
VI	Amyloid beta A4 protein	3.57	4.88E-02	+	A0A067QWW4
VI	Capsid protein	4.03	4.71E-03	+	D9ZD21
VI	C-type-lectin-like-4 protein	5.72	1.47E-04	+	W6MNG5
**Cond.: I vs R**					
I	Aminopeptidase N	-5.98	2.78E-04	−	A0A067QSR5
I	Gamma-crystallin A	-4.37	5.88E-05	−	D3PIA3
I	TnpC	-4.18	4.96E-02	−	G9HZ26
I	Transposase	-4.15	6.26E-03	−	G8UKJ2
III	Beta-crystallin A1	-10.18	1.35E-19	−	D3PHS5
IV	Farnesoic acid O-methyltransferase	3.39	1.29E-02	+	B8X2Z4
IV	FreD	4.91	1.34E-04	+	A0A068LKH5
IV	Leukocyte elastase inhibitor	8.95	2.67E-06	+	G7Y5W4
**Cond.: M vs R**					
I	C-type lysozyme	5.85	3.23E-03	+	B2R4C5
I	Putative nuclease HARBI1	3.44	2.96E-02	+	A0A067RIF9
II	Beta-2-microglobulin	7.57	4.28E-02	+	P16213
II	Elongation factor 1-alpha	8.27	3.23E-03	+	W5PHA3
II	Prosaposin	8.45	1.67E-03	+	A0A024QZQ2
V	Vitellogenin	-3.27	2.96E-02	−	Q9UAR3
V	Capsid protein	-3.77	1.25E-02	−	E1CI71
V	C-type-lectin-like-4 protein	-6.31	9.43E-04	−	W6MNG5
VI	RNA-dependent RNA polymerase	-6.05	2.79E-03	−	A0A023VRY1

Note: Cond.: condition; I: immature; M: mature; R: maturing; Log2FC: Log2FoldChange; P_adj_: adjusted P-value;

−: down-regulated;

+: up-regulated.

**Fig 9:**
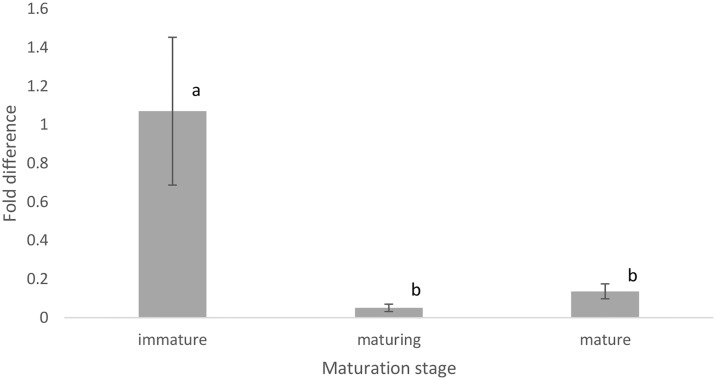
Fold difference of beta crystallin like gene in different maturation stages of testis of male *S*. *olivacea*. 18S rRNA was used as reference gene. Different superscript letters within the same row indicate significant differences (P < 0.0001) between different stages of maturation.

The most significant differentially expressed gene—the 1515 bp beta crystallin like gene (accession no: GDRN01147796.1) was up-regulated in immature specimens but down-regulated in maturing and mature specimens. No significantly differentially expressed genes with minimum threshold of Padj < 1e-10 were found when comparing mature and maturing specimens. The beta crystallin like gene was validated using qPCR and gene-specific primers (Fig 9).

## Discussion

In recent years, the usage of high-throughput sequencing technique to reveal various genomic and genetic information, even in non-model organisms has been steadily gaining momentum [33,34]. In addition, transcriptome sequencing allows the profiling of genes that are differentially expressed under different physiological conditions [35]. Current study used pooled samples to represent each developmental stage for the differential expression analysis as we were interested in the gene expression among stages rather than the inter-individual variation within specific stage. Thus, in this context, pooling minimizes the effects of biological variation (difference among individuals) [36] and highlights the substantive gene expressions expressed during each stage [37]. Konczal et al. [38] reported that when liver transcriptomes of bank voles were sequenced individually and as pooled samples, the accuracy of allele frequency estimation was minimally affected by inter-individual variation in gene expression and that pooled RNA-seq is as accurate as pooled genome resequencing. A total of 17 Gbp transcriptome data consisting of 144,560,704 clean reads were successfully obtained in three runs in present study. The amount of clean reads retrieved were higher than that acquired from the Chinese mitten crab (*Eriocheir sinensis*, 25,698,778 reads in two runs) [12] and boreal spider crab (*Hyas araneus*, 98,508,658 reads in six runs) [39] but lower than that of whiteleg shrimp (*Litopenaeus vannamei*, 399,056,712 reads in four runs) [40]. The average size of assembled transcripts was 886 bp, larger than those found in *E*. *sinensis* (average 191 bp) [12], *H*. *araneus* (average 195 bp) [39] but smaller than the average size of assembled transcripts in *L*. *vannamei* (1137 bp) [40]. Comparably, a transcriptome analysis of a close relative to *S*. *olivacea*, i.e. the yellow mud crab (*S*. *paramamosain*) by 454 deep sequencing generated lesser clean reads (1,314,101 high quality reads) with a smaller average size (411 bp) [41].

The discoveries and annotations of known genes were based on four protein databases, i.e. UniProt, Interpro, Pfam and *D*. *melanogaster* protein database. The low number of successful gene annotations (approximately 15–23% hits when compared to the four protein databases) might be due to unavailability of whole genome of the studied crab species and the scarcity of genomic data of closely related organisms in public domains [41]. Using the same next-gene sequencing (NGS) technology, i.e. Illumina HiSeq 2000 platform, approximately 18.62% of clean reads of a non-model organism, the swimming crab (*Portunus trituberculatus*) were annotated in Swiss-prot [21]. In addition, aquaculture sector and researchers also focus more on female candidates of most commercially important species, resulting in richer genetic information compared to males. The high percentage of unannotated sequences (more than 75%) from the transcriptome data of *S*. *olivacea* implies that potentially useful genetic information, especially differentially expressed genes that might be available was missed and remain unexploited. Thus, current transcriptome data might still hold many important genes and valuable genetic information that can be mined in the near future.

In the transcriptome data of *S*. *olivacea*, predominant gene clusters were found to be involved in various biological processes (e.g. DNA transcription and signal transduction processes) and molecular functions (e.g. molecular binding activities), in addition to formation of structural component of cells, such as nucleus, membrane and cytoplasm. The consistency of gene distribution based on GO terms and GO categories in the present study with other studies [41–43] showed that genes encoding these functions are rather conserved and easily annotatable from database. Functional annotation and enrichment analysis of GO functions aid in mapping out genes and their potential functions at transcriptomic level. The transcriptome data in present study represents an extensive gene catalog particularly expressed in the testis of *S*. *olivacea*, with important role in several biochemical processes such as reproductive development, growth and sexual differentiation. These transcriptome data will be useful for future genomic and gene functional analysis of *S*. *olivacea*.

Although the role of gonad in regulating developmental processes in crustaceans with the aid of a variety of regulatory factors (e.g. hormones and neurotransmitters) have been extensively studied [44–46], the underlying molecular mechanisms governing their biosynthesis remain largely unexplored [21]. Gene sequences related to growth, sexual differentiation and reproduction were identified in the transcriptome data of *S*. *olivacea*. Known for their regulatory role in reproduction in crustaceans [44,47], the identification of crustacean hyperglycemic hormone (CHH) family peptides (Table 6) in this study may aid in providing possible alternatives to the conventional eyestalk ablation methods to promote growth and sexual maturation.

Found in our gonad transcriptome of male *S*. *olivacea*, neurotrophins (Table 6) are vital neurohormones that promote the survival, development and function of neuronal cells [48]. Initially being considered as a characteristic of vertebrates, neurotrophins and their receptors were found in invertebrate crustacean *Daphnia pulex* in the year 2011 [49]. In addition, their roles in testicular development were supported by the findings of the expression of neurotrophins and their receptors in testes of vertebrates [50,51]. It was hypothesized that neurotrophins might be involved in the regulation of male germ cell differentiation via paracrine signalling based on their (neurotrophins and their receptors) different cellular localizations [52].

Identified mostly in insects, neuroparsins are multifunctional neurohormones that are anti-gonadotropic, involved in the regulation of hemolymph lipid and trehalose levels, and in their reproduction development [53,54]. Recently, a crustacean neuroparsin–*Metapenaeus ensis* neuroparsin (*MeNPLP*) homologous to the insect neuroparsin was discovered in most major organs of sand shrimp *Metapenaeus ensis*, including in the hepatopancreas, nerve cord, brain, heart, ovary and muscle. Surprisingly, no expression of *MeNPLP* was found in the testis. *MeNPLP* is involved in the ovarian maturation in shrimp as a drop in the production of vitellogenin protein in hemolymph and ovary was observed following the RNAi silencing of *MeNPLP* [55]. The discovery of neuroparsin gene expression in the testis of *S*. *olivacea* (Table 6) might indicates that the neuroparsin is vital for the development and reproduction of male mud crab but not in shrimp. Similar postulate was proposed to explain the absence of neuroparsin gene in the widely-studied *Drosophila melanogaster* (Arthropoda, Insecta) genome and that due to different metamorphosis patterns, neuroparsin becomes non-essential in some *Drosophila* species [56].

VASA gene is vital for germ cell development, proliferation and maintenance and can be found in both invertebrates and vertebrates [57,58]. This gene encodes for RNA-dependent helicase and is specifically expressed in germ cells throughout all developmental stages [59]. The function and regulation of VASA proteins during gonadal development and gametogenesis have been described for several crustacean species [57,59–62], including *S*. *paramamosain* [31]. Only found to be expressed in the ovary and testis, VASA gene was highly expressed during early gametogenesis of *S*. *paramamosain*, with significantly higher expression levels were observed in the testes of immature and maturing males. In contrary, no significant decrease in the expression of VASA gene was found among different developmental stages of *S*. *olivacea* (Table 6, S2 Appendix). This inconsistency of VASA expression was also found in other crustacean species. For example, in Chinese white shrimp *Fenneropenaeus chinensis*, the expression of VASA gene showed a decrease pattern from spermatogonia to spermatids, and no expression was observed in mature sperm [63]; while VASA RNA was found in the nucleus and cytoplasm of sperms of giant freshwater prawn (*Macrobrachium rosenbergii*) [60].

Prostaglandins (PGs) are cell-signalling autocoids derived from lipids and some are known to be involved in the reproduction development in crustacean, i.e. the level of PGD2, PGE2 and PGF2 α increased with the progression of vitellogenesis and ovarian developmental stages [21,63]. However, most of the previous studies in crustaceans focused on the involvement of PGs on oogenesis and ovarian development [64–66]. In the present study, we identified two PGs, namely PGE2 and PGF (Table 6). Both PGs are known for their regulatory roles during oocyte maturation in animals including crustaceans [64,67,68]. Thus, the findings in this study suggest that PGs might also be involved in the regulation of testicular development in *S*. *olivacea*.

Ecdysteroid receptors (EcR) are nuclear receptors that are to be bound and activated by ecdysteroids [69]. They act as ligand-dependent ecdysteroid signalling mediators and upon binding with ecdysteroids, corresponding genes will be actively transcribed and a cascade reaction will be initiated. Although present in all arthropods, the number of hormones and receptor isoforms’ structures in crustaceans differ with that of insects’. In crustaceans, ecdysteroids are produced by Y-organs and positively regulate molting, gametogenesis and gonad maturation [21,70]. Some EcR splice variants are organ-specific and they might play different roles although present in both sexes [71]. Four types of EcR were found in this transcriptome, namely EcR, EcR2, EcR3 and putative ecdysteroids/dopamine receptor (Table 6). As shown by Li et al. [72] in *Drosophila*, EcRs found in the testis of *S*. *olivacea* might also play the same role—maintenance of testis stem cells.

SNPs are potential markers that are frequently used in trait-mapping and whole-genome association studies due to their wide distribution and abundant polymorphisms [73,74]. They serve as potential markers in non-model species lacking full annotated genome sequences [16,75,76]. For example, an ATP-dependent DNA helicase gene, *RuvB-like 2*, with three SNPs (one exonic and two intronic) was significantly expressed in the ovaries of mature giant tiger shrimp (*Penaeus monodon*) and influenced overall body weight during ovarian development [77]. In addition, four intronic SNPs in the actin and CHH were reported to influence the growth performance in *M*. *rosenbergii* [78]. The mean Ts: Tv ratio (2.22: 1.00) of SNPs reported in current study can aid in the identification of genes affected by selection [76,79]. Studies showed that unlike in fish [75, 80–82], the mean Ts: Tv ratio is species-specific in crustaceans. The mean Ts: Tv ratios in *M*. *rosenbergii* [83] *P*. *trituberculatus* [84], green mud crab (*S*. *paramamosain*) [20] and Chinese mitten crab (*Eriocheir sinensis*) [85] were 1.99: 1.00, 1.00: 1.79, 3.48: 1.00 and 1.00: 1.84, respectively. In addition, the superiority of Illumina HiSeq 2000 over the Roche/454 platform and its potential in the development of SNP markers were highlighted in this study, with approximately eleven-fold increase in the SNPs discovery (13,271 SNPs detected in the testis and ovary of *S*. *paramamosain* as reported by Gao et al. [20] in comparison with 156,181 SNPs found in the testis of *S*. *olivacea* in this study). The putative SNPs found in this study are useful in various fields of fisheries and aquaculture regarding *S*. *olivacea*, such as the study of population genetic structures, conservation of wild population, mapping of economically important traits, and provide resource for potential valuable markers for future selective breeding of *S*. *olivacea*.

The availability of transcriptomic data from the testis of *S*. *olivacea* found in this study proved to be beneficial, in which soon after approximately 160,000 transcriptome shotgun assembly sequences of *S*. *olivacea* were made public in GenBank, our data were mined for putative peptide-encoding transcripts to further understand the peptidergic control systems in *S*. *olivacea* and subsequently suggest possible endocrine manipulation to improve its aquaculture production [86]. Being the largest and most diverse class of hormones, peptides function as major signal transducers and essentially regulate behavioural and physiological changes in all aspects, including growth, sexual development, reproduction and metabolism [87–90]. This mined peptidome identified 49 transcripts encoding putative peptide precursors and subsequently predicted 187 distinct peptides for *S*. *olivacea* [86]. Based on the high similarity in peptide structure and the numbers of peptide families found between *S*. *olivacea* and *S*. *paramamosain* [91], Christie [86] postulated that the physiological roles of these peptides might be conserved in both *Scylla* species. The precursors of neuropeptides found in this study, e.g. CHH and vitellogenin-inhiting hormone (VIH), are mainly produced by the X-organ-sinus gland complex located at the eyestalk ganglia of *S*. *olivacea* [45,92,93]. However, some of the peptide groups, such as the CHH, were reported to be produced and released by non-neuronal tissues (epithelial endocrine cells of the gut) as well in other crab species for the regulation of water and ion during moulting [93]. Thus, the discovery of these putative peptide-encoding transcripts in the testis of *S*. *olivacea* suggests that testis might be involved in the production and regulation of reproductive hormones in *Scylla* spp. and possibly also in other brachyurans or crustaceans more than what we expected. In support of this postulate, a neuropeptide—pigment dispersing hormone (PDH)-encoding transcript, was also found to be produced in the reproductive organs (i.e. ovaries) of *S*. *paramamosain* [94].

The reproductive regulatory mechanism and development are complex processes, with testis being the main regulator. The differentially expressed genes found between the testis expression profile of different maturation stages serve as a large candidate database for the mining of novel genes involved in the gonad development, maturation and reproduction in *S*. *olivacea* and other crustaceans as more than half (65.5%) of the differentially expressed genes are novel genes (S2 Appendix). Most of the annotated differentially expressed genes (e.g. transposase, prosaposin and aminopeptidase) are involved in general cell regulation and signalling pathways (Table 8). Genes that regulate growth, maturation and reproduction such as Farnesoic_acid O-methyltransferase and vitellogenin increased in expression in the testis of *S*. *olivacea* as the crab matures. Other genes expressed in testis such as *Dmrt* (reported in *S*. *paramamosain* [20] and *E*. *sinensis* [95]) and Feminization-1 (*FEM-1*) (reported in *S*. *paramamosain* [20]) that are involved in sex differentiation and testis development were not found in this study. Of a total of 200 genes that were differentially expressed at Padj < 0.05 (S2 Appendix), beta crystallin like gene was the most significant differentially expressed gene at Padj < 1e-10 (up-regulated in immature stage but down-regulated in maturing and mature stages). Thus, this gene serves as a good candidate for a marker of immaturity in crab testis. The beta crystallin domain (Pfam PF00030) is a water-soluble calcium binding domain found in a diverse set of proteins. Proteins within this domain are multifunctional and although primarily found in the eye lens, beta crystallin is also regulated in other sites such as brain and testis [96,97]. Found in all vertebrate classes, beta crystallin is highly expressed during developmental stages, presumably involved in the formation of complex optical properties in the eye lens [98]. In addition, betaB2-crystallin proteins are postulated to be involved in fertility as mutation in betaB2-crytallin gene resulted in subfertile mice in both males and females [99,100]. This gene is found to be upregulated in the testis of mice during the initiation of spermatogenesis [99], similar to the result found in current study. The relationship between beta crytallin proteins and the gonad maturation in invertebrates is still unexplored and this study serves as the first report of this gene in invertebrate and its possible involvement in the gonadal maturation. This finding broadens our understanding on the reproductive biology of invertebrates, particularly crustaceans, as they are known for regulating their reproductive development with the aid of neuropeptides produced in the eyestalk [45]. If their functions remain the same, the beta crystallin like proteins are also likely to be found in the eye lens of crustaceans. Thus, the use of the frequently adopted procedure of eyestalk ablation to promote faster gonadal maturation especially in male crustaceans for aquaculture production need to be reviewed because although eyestalk ablation removes testis inhibiting factors and resulted in the increase in the size of testis and the number of number of mature spermatocytes [101,102], it also removes beta crystallin like proteins, which promotes testicular maturation and the absence of it may influence fertility. The negative effect of eyestalk ablation on the quantity and quality of spawning, and subsequent larvae viability have been reported in female crustaceans [103,104]. Further study on this specific beta crystallin like gene that was found upregulated in immature male *S*. *olivacea* might provide more insight on its involvement in crustacean fertility and reproductive development.

## Conclusions

The first transcriptome analysis on the testis of orange mud crab (*S*. *olivacea*) was carried out successfully and yielded 144,560,704 high quality reads. Present study also demonstrated the usefulness of next generation sequencing (Illumina) in characterizing transcriptome profile and gene expression of non-model organism using tissue-specific samples. Data obtained in present study greatly contributes to the understanding of the gene expression and genome structure occurring within the testis of *S*. *olivacea* throughout its developmental stages. Potential SNPs reported in this study is useful for future selective breeding, trait-mapping, and gene localization studies. The discovery and validation of differentially expressed beta crystallin like gene based on the testis transcriptome profiles of *S*. *olivacea* show that this particular gene might be suitable to be use as immaturity marker in male *S*. *olivacea* in the future.

## Supporting Information

S1 Appendix Potential SNPs. (XLSX)

S2 Appendix Up- and down-regulated genes. (XLSX)
